# Proposal of an obesity classification based on weight history: an official document by the Brazilian Society of Endocrinology and Metabolism (SBEM) and the Brazilian Society for the Study of Obesity and Metabolic Syndrome (Abeso)

**DOI:** 10.20945/2359-3997000000501

**Published:** 2022-07-01

DOI: 10.20945/2359-3997000000465

Arch Endocrinol Metab. 2022;66(2):139-51

Where you read:

**Table 2.** Newly proposed classification based on two clinical cases[Table t1][Table t2]

**Case 1: t1:** A 55-year-old man with a maximum weight achieved in life (MWAL) 2 years earlier of 118 kg and a height of 175 cm (BMI of 35.9 kg/m^2^).

Hypothetical scenarios	Weight (kg)	BMI (kg/m^2^)	Percentage of weight loss based on MWAL	Newly proposed classification	Traditional classification
1A	115	38.5	2.5%	Class II obesity (unchanged)	Class II obesity
1B	108	35.2	8.4%	Class II obesity (5% reduced)	Class II obesity
1C	103	33.6	12.7%	Class II obesity (10% controlled)	Class I obesity
1D	98	32.0	16.9%	Class II obesity (15% controlled)	Class I obesity

**Case 2: t2:** A 40-year-old woman with an MWAL of 100 kg (6 months earlier) and a height of 156 cm (BMI 41 kgm^2^).

Hypothetical scenarios	Weight (kg)	BMI (kg/m^2^)	Percentage of weight loss based on MWAL	Newly proposed classification	Traditional classification
2A	98	41	2%	Class III obesity (unchanged)	Class III obesity
2B	94	39.1	6%	Class III obesity (unchanged)	Class II obesity
2C	89	37	11%	Class III obesity (10% reduced)	Class II obesity
2D	82	34.1	18%	Class III obesity (15% controlled)	Class I obesity

Should read:

**Table 2.** Newly proposed classification based on two clinical cases[Table t3][Table t4]

**Case 1: t3:** A 55-year-old man with a maximum weight achieved in life (MWAL) 2 years earlier of 118 kg and a height of 175 cm (BMI of 38.5 kg/m^2^).

Hypothetical scenarios	Weight (kg)	BMI (kg/m^2^)	Percentage of weight loss based on MWAL	Newly proposed classification	Traditional classification
1A	115	37.5	2.5%	Class II obesity (unchanged)	Class II obesity
1B	108	35.2	8.4%	Class II obesity (5% reduced)	Class II obesity
1C	103	33.6	12.7%	Class II obesity (10% controlled)	Class I obesity
1D	98	32.0	16.9%	Class II obesity (15% controlled)	Class I obesity

**Case 2: t4:** A 40-year-old woman with an MWAL of 100 kg (6 months earlier) and a height of 156 cm (BMI 41 kgm^2^).

Hypothetical scenarios	Weight (kg)	BMI (kg/m^2^)	Percentage of weight loss based on MWAL	Newly proposed classification	Traditional classification
2A	98	40.2	2%	Class III obesity (unchanged)	Class III obesity
2B	94	38.6	6%	Class III obesity (unchanged)	Class II obesity
2C	89	36.5	11%	Class III obesity (10% reduced)	Class II obesity
2D	82	33.6	18%	Class III obesity (15% controlled)	Class I obesity

